# ViralWasm: a client-side user-friendly web application suite for viral genomics

**DOI:** 10.1093/bioinformatics/btae018

**Published:** 2024-01-10

**Authors:** Daniel Ji, Robert Aboukhalil, Niema Moshiri

**Affiliations:** Department of Computer Science & Engineering, UC San Diego, La Jolla, CA 92093, United States; Chan Zuckerberg Initiative, Redwood City, CA 94063, United States; Department of Computer Science & Engineering, UC San Diego, La Jolla, CA 92093, United States

## Abstract

**Motivation:**

The genomic surveillance of viral pathogens such as SARS-CoV-2 and HIV-1 has been critical to modern epidemiology and public health, but the use of sequence analysis pipelines requires computational expertise, and web-based platforms require sending potentially sensitive raw sequence data to remote servers.

**Results:**

We introduce ViralWasm, a user-friendly graphical web application suite for viral genomics. All ViralWasm tools utilize WebAssembly to execute the original command line tools client-side directly in the web browser without any user setup, with a cost of just 2-3x slowdown with respect to their command line counterparts.

**Availability and implementation:**

The ViralWasm tool suite can be accessed at: https://niema-lab.github.io/ViralWasm

## 1 Introduction

The genomic surveillance of viral pathogens such as SARS-CoV-2 and HIV-1 has been critical to modern epidemiology and public health (Robishaw *et al*., 2021). Reconstructing viral genome sequences from samples collected from patients typically requires the use of sequence analysis pipelines such as iVar (Grubaugh *et al.* 2019), HAPHPIPE (Gibson *et al.* 2020), HAVoC (Truong Nguyen *et al.* 2021), V-pipe (Posada-Céspedes *et al.* 2021), VGEA (Oluniyi *et al.* 2021), and nf-core/viralrecon (Ewels *et al.* 2020). However, the use of such pipelines typically requires computational expertise to be able to install and execute command line tools. Alternatively, researchers are able to use web-based platforms such as Galaxy (The Galaxy Community et al. 2022) and Genome Detective (Vilsker *et al.* 2019), but use of these systems requires sending the raw sequence data (which may contain sensitive host contamination from the patient) to remote servers, which can be problematic with respect to patient privacy and HIPAA compliance (Banimfreg 2023).

As web browsers have become increasingly sophisticated, tool developers have been given the ability to write complex web applications that are able to run client-side, directly in the user’s web browser. One such advancement was the development of WebAssembly, which is a low-level bytecode into which native C/C++ code can be compiled and that aims to be safe, fast, portable, and compact (Haas *et al.* 2017), albeit with a slight performance cost with respect to native x86 code (Spies and Mock 2021). As a result, standard command line tools can be compiled into WebAssembly and incorporated into JavaScript web applications in which the WebAssembly modules are initialized once the user visits the website, without any user intervention, and executed directly in the web browser, with little to no modification of the original source code.

Here, we introduce ViralWasm, a client-side user-friendly graphical web application suite for viral genomics. Unlike existing viral genomics pipelines, ViralWasm tools require no setup whatsoever: once the user navigates to the ViralWasm website, all dependencies are loaded automatically as WebAssembly modules. Further, unlike existing pipelines, ViralWasm has an intuitive Graphical User Interface (GUI) with reasonable default values and examples. Lastly, and critically for sensitive patient data, ViralWasm runs completely client-side on the user’s own machine (ie, the user’s data are not sent anywhere), and it can even be run offline easily on Windows, Mac OS X, and Linux.

## 2 Methods

All ViralWasm tools utilize WebAssembly to execute the original command line tools client-side directly in the web browser. We use biowasm (biowasm.com) to load C and C++ tools, and we use Pyodide (pyodide.org) to load Python tools. Biowasm is a repository of recipes for compiling popular C/C++ bioinformatics tools to WebAssembly and provides pre-compiled WebAssembly binaries for use in web applications. The Aioli library provides JavaScript utilities for managing these WebAssembly modules and runs them in a background thread (WebWorkers) in the browser to maintain a responsive UI. As part of this project, we added recipes to biowasm for tn93, ViralConsensus, and FastTree so that other developers can use these tools in their own web applications as well. The GUIs of all tools utilize Sassy CSS and Bootstrap for aesthetics. All testing and benchmarking is performed in a “Continuous Integration/Continuous Delivery” fashion using GitHub Actions, running Playwright tests on an Ubuntu 22.04 environment. The web applications themselves are deployed using GitHub Pages. We also utilize the Pako Javascript package in order to compress user-loaded files to memory before running the pipelines, and we use the Marked JavaScript package to parse Markdown for display on the websites. To run the web applications in offline mode, we provide a Python script that starts a local multithreaded HTTP server.

## 3 Results

The ViralWasm application suite consists of a main landing page (niema-lab.github.io/ViralWasm) that navigates users to the core pipelines (ViralWasm-Consensus and ViralWasm-Epi) and standalone tool web applications. For the sake of reproducibility, ViralWasm web applications specify the versions of all Bioinformatics tools they utilize, and all web applications can be downloaded as a zip archive for local deployment on the user’s machine. For the sake of security, these locally downloaded archives can be deployed completely offline. For the sake of education, ViralWasm tools print to the log the exact commands that would be executed to run each tool from the command line, meaning ViralWasm can be used as an instructional tool to learn how to run Bioinformatics workflows from the command line.

### 3.1 ViralWasm-Consensus

**Figure 1.**
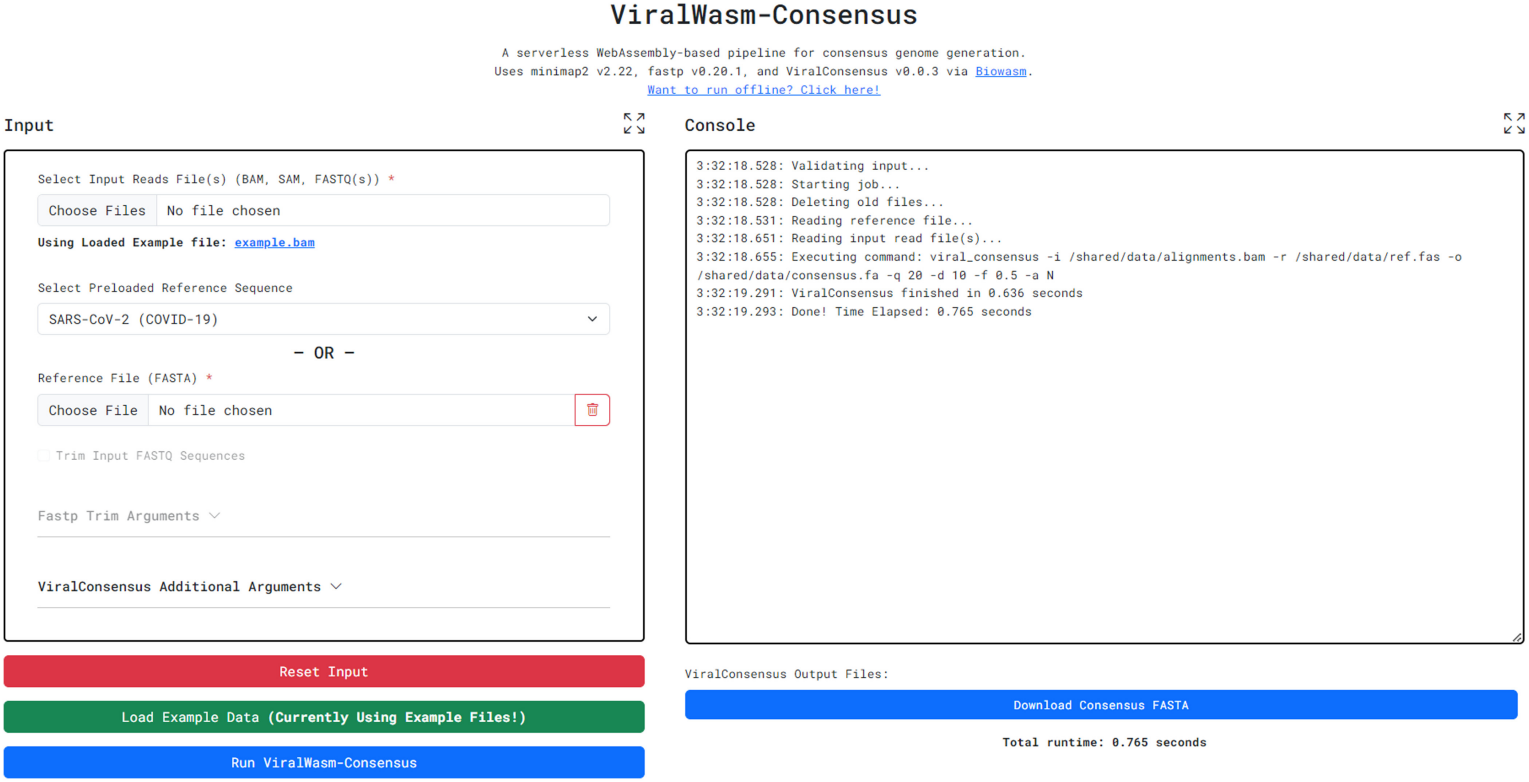
ViralWasm-Consensus. Screenshot of an example ViralWasm-Consensus run.

ViralWasm-Consensus (niema-lab.github.io/ViralWasm-Consensus) is a pipeline for consensus sequence generation (Fig. 1). As input, users provide (i) viral reads in the FASTQ, SAM, or BAM file formats and (ii) a reference genome in the FASTA format or select one from a preloaded set of popular viral reference genomes.

If the user provides a FASTQ file as input, the reads are mapped against the user-selected reference genome using Minimap2 (Li 2018) using its default mapping parameters. Optionally, users can choose to first trim the reads using fastp (Chen *et al.* 2018). For the sake of simplicity, we currently only support trimming a user-specified number of bases from the front and/or tail of reads, polyG tail trimming, and 3’ polyX trimming, but any fastp parameters can be added in the future.

A consensus viral genome sequence is then constructed from the SAM/BAM using ViralConsensus (Moshiri 2023). Sensible parameter values are selected by default, but all ViralConsensus parameters can be overridden as desired by the user (e.g. to increase or decrease strictness).

Once the execution has completed, the user can download the resulting consensus sequence in the FASTA file format, and the user can also optionally download any other output files that were produced during the execution of the pipeline (e.g. base and insertion counts produced by ViralConsensus, the SAM file produced by Minimap2, and the trimmed FASTQ files produced by fastp).

### 3.2 ViralWasm-Epi

**Figure 2.**
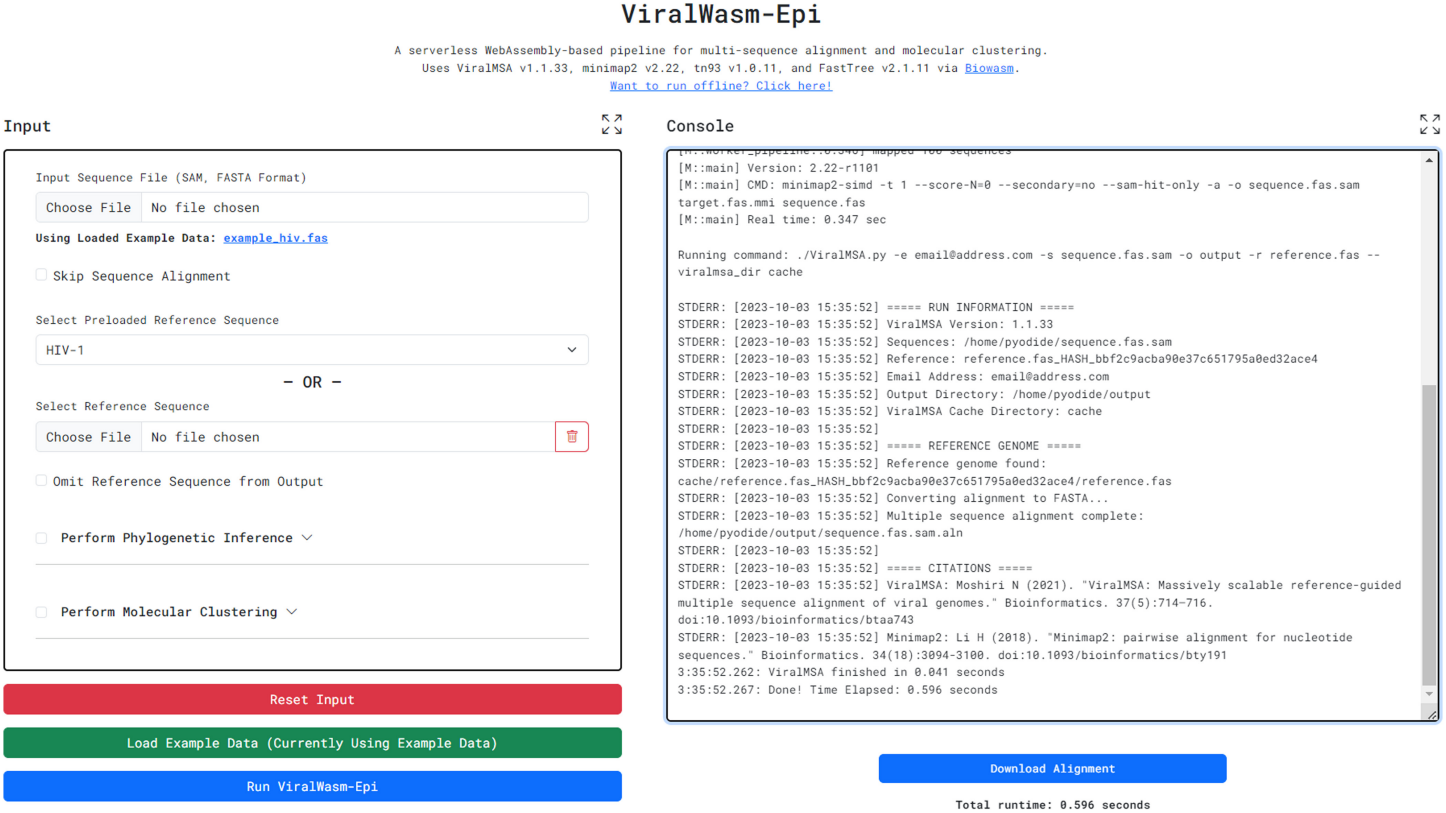
ViralWasm-Epi. Screenshot of an example ViralWasm-Epi run.

ViralWasm-Epi (niema-lab.github.io/ViralWasm-Epi) is a pipeline for viral molecular epidemiology (Fig. 2). As input, users provide (i) viral genome sequences in the FASTA file format, (ii) a reference genome in the FASTA format or select one from a preloaded set of popular viral reference genomes, and optionally (iii) a plain-text file containing sample collection dates.

If the user provides an unaligned FASTA file as input, multiple sequence alignment is performed using ViralMSA (Moshiri 2021). The user can then choose to perform phylogenetic inference using FastTree 2 (Price *et al.* 2010), phylogenetic rooting and dating using LSD2 (To *et al.* 2016), and/or single-linkage genetic distance molecular clustering under the Tamura-Nei 93 (TN93) substitution model (Tamura and Nei 1993) using the tn93 tool from HIV-TRACE (Kosakovsky Pond *et al.* 2018). All FastTree 2, LSD2, and tn93 parameters can be adjusted from their default values by the user as desired.

Once the execution has completed, the user can download any output files (e.g. the FASTA file produced by ViralMSA, the distances TSV file produced by tn93, the clusters TSV file produced by ViralWasm-Epi, and the Newick tree files produced by FastTree 2 and LSD2).

### 3.3 Standalone web applications

In addition to ViralWasm-Consensus and ViralWasm-Epi, which are intended to be complete end-to-end pipelines for their respective analyses, the ViralWasm application suite also provides standalone web applications for each individual tool utilized by either pipeline, as well as for other Bioinformatics tools that may be of interest for viral analyses. Currently, ViralWasm contains standalone web applications for the tn93 tool from HIV-TRACE, ViralConsensus, and ViralMSA.

### 3.4 Benchmarking

ViralWasm-Consensus and ViralWasm-Epi were benchmarked against their exact command line counterparts. ViralWasm-Consensus was benchmarked using a SARS-CoV-2 Illumina amplicon sequencing dataset that was subsampled to various numbers of reads (Moshiri *et al.* 2022), and ViralWasm-Epi was benchmarked using a dataset of full HIV-1 genome sequences obtained from NCBI Virus. All benchmarks were performed via GitHub Actions using the default configuration, which runs in an Ubuntu 22.04 environment running on Azure Standard_DS2_v2 virtual machines with 2 Intel(R) Xeon(R) CPU E5-2673 v3 @ 2.40 GHz CPUs and 7GB of RAM. Command line tools were compiled and installed from source, and ViralWasm tools were executed using Playwright (playwright.dev).

**Figure 3.**
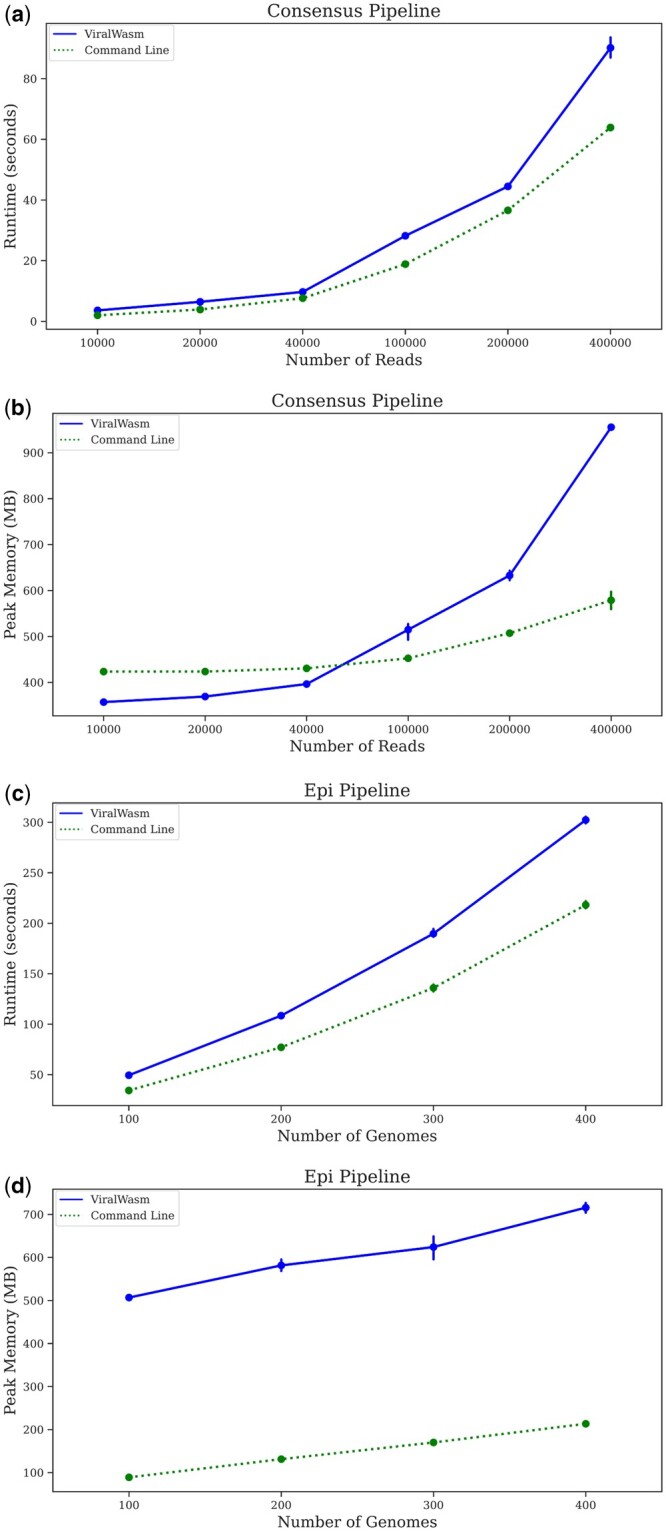
Benchmark results. Runtime and peak memory usage for ViralWasm and its command line counterparts for the Consensus (a, b) and Epi (c, d) pipelines for various dataset sizes.

As can be seen in Fig. 3, the ViralWasm-Consensus and ViralWasm-Epi pipelines are slightly slower and have higher peak memory usage than their command line counterparts. This is expected: by compiling the tools into WebAssembly rather than native code, ViralWasm gains convenience and ease-of-use at the expense of performance. However, both pipelines still run quite quickly with reasonable memory usage on fairly large datasets. ViralWasm-Consensus took roughly 90 s and used roughly 1 GB of memory to run on a dataset containing 400 000 SARS-CoV-2 reads (roughly 2000× coverage), well above what is needed for accurate consensus sequence calls from amplicon sequencing data (Moshiri 2023). ViralWasm-Epi took roughly 300 s and used roughly 700 MB of memory to run on a dataset containing 400 complete HIV-1 genome sequences, which is a reasonable size for real-world molecular epidemiological studies (Little *et al.* 2014).
